# Public-private partnership (3Ps) in ensuring safe use of medicines: An Indian experience

**DOI:** 10.3389/fpubh.2022.930696

**Published:** 2022-08-11

**Authors:** Vivekanandan Kalaiselvan, Shatrunajay Shukla, Shubhang Arora, Tarani Prakash Shrivastava, Rajeev Singh Raghuvanshi

**Affiliations:** ^1^Indian Pharmacopoeia Commission, Ministry of Health and Family Welfare, Government of India, Ghaziabad, India; ^2^Hospital Administration & Management, Yashoda Super Speciality Hospital, Ghaziabad, India; ^3^Department of Pharmacology, Delhi Pharmaceutical Sciences and Research University, New Delhi, India

**Keywords:** pharmacovigilance, public-private partnership, Individual Case Safety Reports (ICSRs), drug safety, Adverse drug reactions (ADRs)

## Abstract

Adverse drug reactions (ADRs) are major concerns to the public health. To monitor ADRs and ensure patients' safety, the Pharmacovigilance Programme of India (PvPI) has been established by the Government of India in 2010. The programme is intact with the Public-Private Partnership (3Ps) in pharmacovigilance for quality services, better management of human resources and risk minimization. The present work is aimed at assessing the 3Ps engagement, performance and tangible outcomes in PvPI and also mapping of resources. The study was carried out for the period of 2011 to 2021 by assessing the various benchmarking tools such as 3Ps categorization, utilization of ADRs reporting tools, trainings, and the Individual Case Safety Reports' (ICSRs) quantity, quality and transmission for regulatory intervention (RI). Under PvPI, Central or State Government medical institutions/hospitals and public health programmes constitute public partners while private medical institutions/hospitals, pharmaceutical companies, corporate hospitals and professional bodies account for private partners. We observed that public partners extensively used ADR reporting form and toll-free helpline number while private partners used mobile based app and emails/post as preferred tools for reporting ADRs. Contribution of public sector in training programmes organized, stakeholders trained and sharing of resource materials was way higher than the private sector. The study revealed that 55.1 and 44.9% ICSRs were received from public and private partners, respectively during the study period. The quality completeness of data received from public partners was found to be 0.92/1 as compared to 0.46/1 from the private partners. The ICSRs data transmitted for RI process from the public and private partners (till 2018) was found to be 79 and 21%, respectively. In terms of sharing of resources for training and capacity building, the public sector played a major role. The 3Ps in India are enabled to establish a robust system for medicines' safety surveillance; however a more focused approach is required in mapping the resources.

## Introduction

Adverse drug reactions (ADRs) are among dominant causes of mortality (1); and expected to pose a great threat to public health in near future (2). Therefore, prompt ADR monitoring and informing the authority for necessary intervention through a pharmacovigilance system is crucial for ensuring drug safety. According to the WHO, Pharmacovigilance (PV) is defined as the science and activities related to the detection, assessment, understanding and prevention of adverse effects or any other drug-related problem (3). The ministry of Health & Family Welfare, Government of India launched its nationwide Pharmacovigilance Programme of India (PvPI) in 2010 and identified Indian Pharmacopeia Commission (IPC) as National Coordination Center (NCC) for the PvPI in 2011 with the aim of monitoring the safety of drugs consumed by the Indian population (4).

The usage of medicines in the private sector is consistently much higher than in the public sector. A Government survey in the year 2014 showed that more than 70% of illnesses were treated in the private sector, including clinics, hospitals and charitable institutions, a four percentage increase over a 10 year period (5). National Sample Survey Office (NSSO) reported that private doctors are the single source of treatment in both the urban and rural areas (5). Also, due to the exponential growth of private pharmaceuticals sector, there is an urgent need to provide handholding support to private sector to meet the challenges such as under reporting of ADRs, development of indigenous patient safety database and for mutual benefits including recognition and to avoid regulatory action (6). Engagement of healthcare professionals is key to success for Pharmacovigilance (7); Therefore, the alliance by the PvPI with public and private hospitals/academic medical institutions has resulted in establishing a robust system and tools for ADRs monitoring, reporting and assessment. In order to ensure seamless ADR-reporting, mobile app and helpline facilities have also been developed besides customized paper-based reporting form (8, 9).

The available scientific articles revealed that public-private partnerships (3Ps) is one of the key components in promotion of healthcare and can also influence intellectuals and policy decisions (10, 11). The 3Ps is also considered to be an important mechanism for ensuring sustainable development goals (12). As PvPI is being successfully implemented with the 3Ps using existing government systems and regulatory framework, wherever possible and aligned with the policies and strategies, the aim of this study is to assess the engagement, performance and tangible outcomes of the ongoing 3Ps activities in ensuring safe use of medicines in India. This will also identify barriers for equitable alignment in technical, policy and mapping of resources.

### Study design

The study was conducted retrospectively for a period of ten years (2011–2021). The public and private partners, those formally recognized as ADR monitoring centers and informally engaged with PvPI for ADR-reporting and capacity-building were categorized accordingly. The training conducted and its outcome, resource-sharing and capacity-building were assessed. The Individual Case Safety Reports (ICSRs) provided by public-private partners were segregated from VigiFlow/VigiBase (a web-based tool owned by WHO-Uppsala Monitoring Center, Sweden and subjected to quantitative and quality completeness assessment. The quality completeness of the ICSRs was assessed as per the method established by Kalaiselvan et al., 2015 (13). The ICSRs of both public and private partners transmitted for regulatory intervention (RI) process were also assessed.

### “3Ps”-categorization and engagements

Since its inception, the PvPI has been consistently engaged in identifying the partnering institutions both from public and private sector. The working relationship was gauzed through either formal Memorandum of Understating (MoU) or mutual understanding to attain common benefits. The present PvPI structure has a mixed set-up: where both the public (Government of India and Government of States and Union Territories) and private academic medical institutions/hospitals as ADR Monitoring Centers (AMCs) pan-India coexist. The 446 AMCs established during the study period were unique in nature and equally distributed in public and private sector; 224 and 222 AMCs in the public and private sector, respectively. The AMCs are primarily responsible for monitoring and reporting ADRs to PvPI. In addition, the PvPI has established a working relationship with other private entities such as Marketing Authorization Holders (MAHs) of pharmaceutical industries, corporate hospitals and professional bodies to attain the common goal of promoting the safety of medicines.

### ADR reporting by “3Ps”-voluntary or mandatory

The private sector i.e., MAHs are mandatorily required to submit the adverse events associated with use of new drugs mandatorily (up to four years from the date of approval) to the National Regulatory Authority i.e., Central Drugs Standard Control Organization (CDSCO). In addition, the CDSCO also encourages MAHs to submit voluntary reports of ICSRs associated with medicines to PvPI. For the last ten years (2011–2021), 130 MAHs of pharmaceutical products have participated in the PvPI by reporting ADRs. However, ADR reporting by recognized AMCs under PvPI is primarily voluntary in nature. The PvPI encourages them as well as other healthcare professionals associated with corporate hospitals/clinics/professional bodies and government hospitals to report ADRs by solicited spontaneous (voluntary) method.

### ADR reporting tools for 3Ps

**Figure 1:**
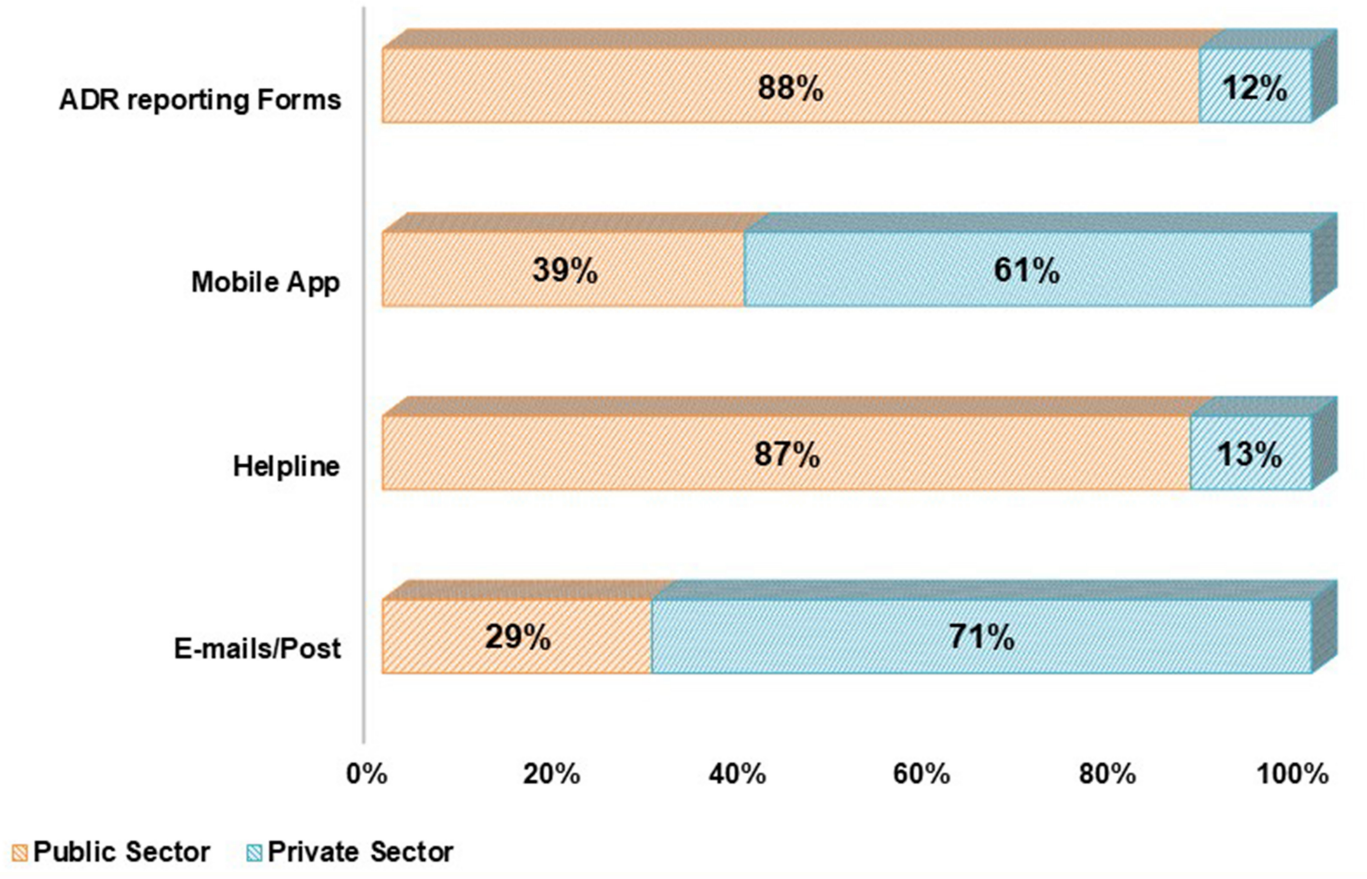
Utilization of adverse drug reactions reporting tools to ensure safe use of medicines by public and private stakeholders under 3Ps.

Public and private partners were provided with multiple tools to report ADRs; the customized reporting form which is paper-based was often recognized as one of the most widely used tools for reporting ADRs but as per the feedback received from the stakeholders, it is a time-consuming tool. In order to overcome the shortcoming and enhance the stakeholders involvement to gain momentum, tools such as Helpline (toll free number 1800 180 3024) and mobile app were developed. Since 2014, PvPI has been accepting the electronic submission of both expedited and non-expedited ICSRs from MAHs in the Extensible Markup Language (XML) as per E2B guidelines (14). This tool was developed by MAHs at their own expenditure. We observed that ADR reporting tools such as paper-based reporting form (also available in various regional languages) and toll-free helpline number were mostly used by the public sector (88 and 87%, respectively). Private sector used mobile-based application and emails/post by 61 and 71% respectively, while reporting ADRs to PvPI (Figure 1).

### Training and capacity building

PvPI appoints a dedicated, qualified and trained human resource team at recognized AMCs (both public and private sector) to foster pharmacovigilance practices. Also, an AMC focal person (A focal person may have a degree in medicine, dentistry, pharmacy, clinical pharmacology, pharmacy practice or clinical research and possesses minimum 1 year experience in drug safety or pharmacovigilance) along with their team were continuously trained and provided with technical guidelines, ADRs reporting tools and other accessories by the PvPI for seamless functioning. All recognized AMCs were enabled to submit the ICSRs through VigiFlow. Besides, PvPI in alliance with MAHs of pharmaceutical products and professionals bodies such as Indian Medical Association, Indian Pharmacological Society and others exhorted them to receive ADRs from their quarters. Under 3Ps, private partners were urged to join for a professional cause to promote drug safety with no commercial interest.

PvPI conducted numerous training programmes during the period of 2011 and 2021 with the support of both public and private sector. The objectives of such training programmes were aimed at providing the basic concepts and understanding of ADR-reporting, tools and methods, causality assessment, regulatory pharmacovigilance and signal detection. The trained participants of public and private sector were geared to propagate the concept and adopt the practice of pharmacovigilance for mutual benefits. The trainings were organized by PvPI alone (managed with its own resources) where healthcare professionals of public and private sector participated or jointly organized by PvPI and private sector where the resources were mapped. During the period 2011–2021, in total, 3,976 trainings (2,468 by public sector and 1,508 by private sector) were conducted under the umbrella of PvPI to clear the concept and provide hands-on training on ADR-monitoring and reporting and translational/regulatory pharmacovigilance. Technical expertise available in both public and private sector was fully utilized in the training programmes. As many as 191,835 healthcare professionals, including doctors, pharmacists, nurses, drug regulators, patients' support-group and others, were trained during the study period. These training programmes were instrumental in enhancing reporting of ADRs. A significant average increase of 59 and 41% in ADR reporting was observed by public and private sector, respectively following training programmes. A contribution of 75 and 25% in publication of scientific articles, reviews and case studies were noted by public and private sector, respectively (Table 1). The major outcomes of these training programmes are development of a sense of responsibility and to raise awareness on what, how and where to report ADRs, and to ensure that mere reporting an ADR does not attract any legal implication for reporter. Taken together, these changes fostered the culture and quality of ADR-reporting in a big way.

**Table 1:** Contribution of public-private partners in knowledge and resource sharing.

**S. No**.	**Indicators**	**Contribution by public sector**	**Contribution by private sector**
1.	Training programmes organized	2,468	1,508
2.	Healthcare professionals trained	126,602	65,233
3.	Impact of training programmes (Average % increase in ADR reporting after every subsequent training)	59%	41%
4.	Sharing of information/resource materials	75%	25%

### 3Ps' data generation-quantity, quality and signal detection

**Figure 2:**
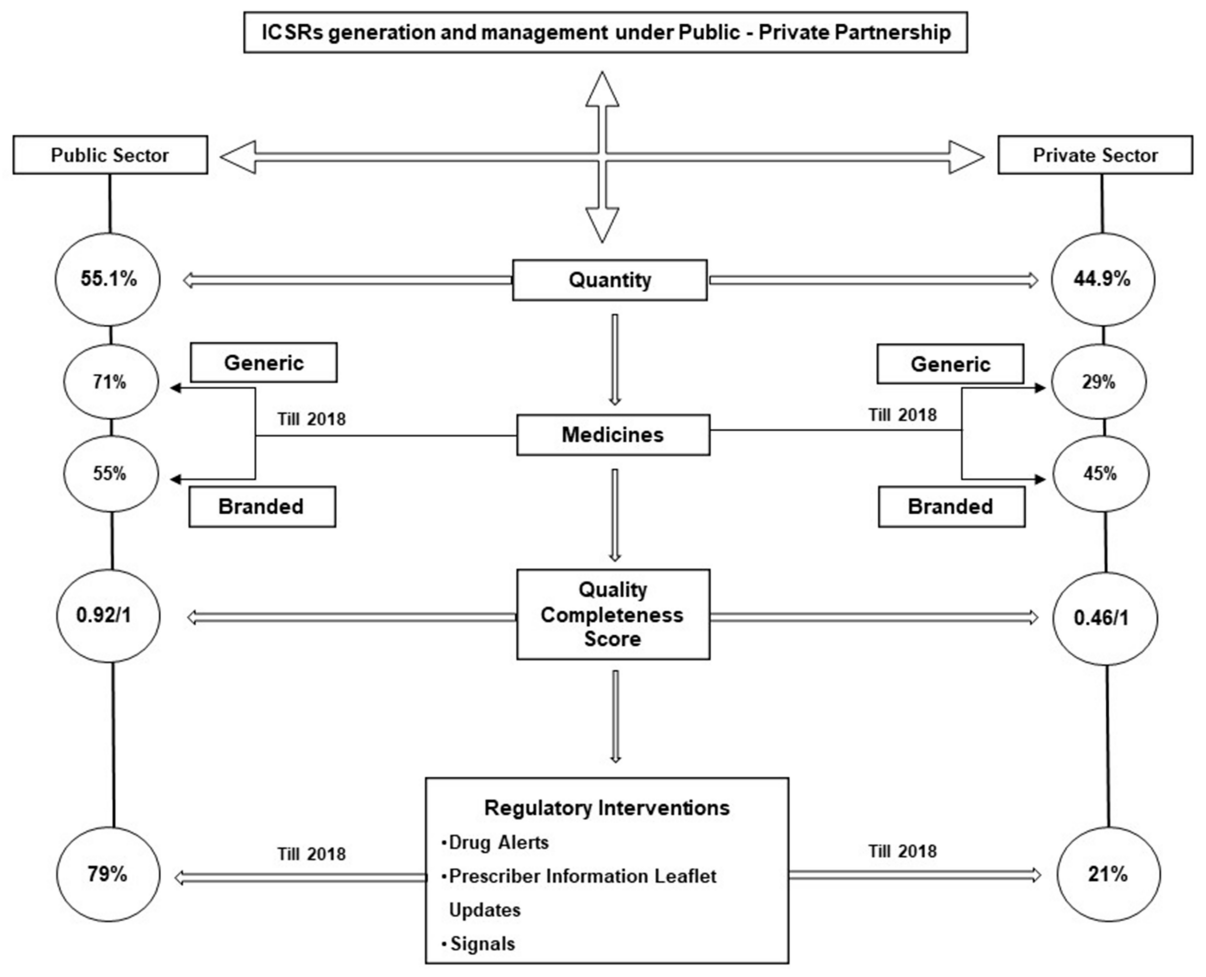
Contribution of public and private stakeholders in individual case safety reports generation, management, quality of reports and regulatory interventions.

For the last ten years 584,015 ICSRs were generated using the 3Ps' approach and assessed by the PvPI, of which 321, 657 (55.1%) were reported by the public AMCs and its periphery public sector (Central, States/UTs) academic institutions/hospitals, public health programmes such as National Tuberculosis Elimination Programme, National AIDS control programme, National Center for Vector Borne Diseases Control (NCVBDC) and Universal Immunization Programme. A total of 262,358 (44.9 %) ICSRs were identified as reported by private sector which includes the AMCs under private ownership and MAHs of pharmaceutical products. Around 71 and 29% of the ICSRs reported by the public and private sector, respectively, accounted for the use of generic medicines and nearly 55 and 45 % of the ICSRs reported by the public sector and private sector, respectively, accounted for branded medicines (till 2018). The quality of ICSRs submitted using 3Ps' approach was assessed during the study period (2011–2021). As per the UMC guidelines, the quality completeness score for ICSRs ranges from 0.07 to 1 (15). The average quality of ICSR data provided by public partners was found to be 0.92 as against 0.46 by the private partners. The quality of data plays an important role in clinical assessment and thus identifying new safety signals. The new signals or meaningful findings thus generated were communicated to CDSCO for making an appropriate regulatory decision. Most of the ICSRs received from the private sector, unlike ICSRs received from the public sector, were lacking in information such as the identifiable reporter, reaction details, laboratory investigation details and causality assessment. The ICSRs contributing to detecting safety signals (a new or unknown ADR, not reported previously) and other RIs from public and private sector have been illustrated in Figure 2.

### 3Ps impact in international arena

The collective approach by 3Ps witnessed a successful National Regulatory Authority (NRA) assessment for vaccines where PvPI played a key role in enhancing the reporting of adverse events associated with vaccines. The 3Ps' contribution helped Indian NRA to get the highest benchmarking score for the tool of Vigilance for the assessment year 2017. As per data available at WHO-Uppsala Monitoring Center, India is one of the top 10 ADR-reporting countries in terms of quantity and highest among all in terms of quality (16, 17). Also, India-specific data contributed to WHO-UMC in detecting the global signals associated with use of drugs and vaccines. The India-specific drug-safety information is regularly published in WHO pharmaceuticals newsletter. By collective efforts and its 3Ps deliverables, the PvPI has been recognized globally by the WHO as one of its Collaborating Centers (WHO-CC) for pharmacovigilance in Public Health Programmes and Regulatory Services in 2017 for 4 years and recognition is further extended to four years i.e. upto 2025.

## Discussion

Though PvPI has played a key role in establishing AMCs in the private sector, the quantity and quality of ICSRs reported by the public sector hospitals during the period 2011–2021 has been appreciably high (18). Aimed at fostering the culture of reporting by 3Ps, the PvPI has contributed by sending circulars, appreciation letters, feedback, etc. As per the feedback received, the reasons for low reporting vary between the public and private sector. More workload or lack of time is the major concern for public sector whereas in the private sector the perception is that reporting of more ADRs may affect the reputation of the hospitals/organization concerned. The PvPI, therefore, addresses the issues by education and advocacy to the hospital management and team of healthcare professionals that ADR-reporting is voluntary in nature entailing no punitive action. Even the quality completeness score of reports received from the public sector is higher than those received from the private sector. The quality score of the reports received from private sector is low as they do not want to reveal complete information of the ADRs, fearing litigation. The number of ICSRs contributing to identifying new signals or update of the package insert has been mostly from the public sector. The public sector contributed significantly higher reporting of ADRs associated with the use of generic as well as branded medicines as compared to the private sector.

The PvPI has played a pivotal role in capacity-building at both public and private sector, enabling the organizations to strengthen their competency in developing and implementing Good Pharmacovigilance Practices (GVPs). The public sector shared/spent around 75% volume of the resource/finance as against the remaining 25% by the private sector. The tailor-made pharmacovigilance training course developed by the PvPI has been effectively utilized to familiarize professionals with the concept of ADR-reporting and management. In terms of participation and organizing the training programmes, both private and public sector have played an equal role. It has been observed that the reporting of ADRs after each level of training has increased exponentially.

The PvPI has successfully attempted to excel the practice of pharmacovigilance with the introduction and promotion of emerging technologies in consonance with International standards. At present, the PvPI operates with full financial support by Ministry of Health & Family Welfare, Government of India. The private sector will require arranging resources for providing training to the stakeholders as the current drug regulation in India makes pharmacovigilance mandatory for MAHs. This will also require private partners to take ownership of the activity. The 3Ps in India have paved the way for generation of nearly 0.58 million ADRs in PvPI database and helped the Indian regulatory authority to be self-reliant in RI process. However, to achieve the sustainable development goal of PvPI, there is an urgent need for developing/leveraging ADR reporting/analyzing technologies such as mobile app, e-reporting and making these enabling tools available to 3Ps in a more effective way which will also help reduce the overall cost of PV system operation (19, 20). Harmonized and partnered approach is essential, too, to save the cost and mapping of resources. The PvPI or public funding alone may not be sufficient to meet the challenges of pharmacovigilance. However, the private sector participation and sharing of the resources for pharmacovigilance needs to be enhanced in future. Though, 3Ps in particular to healthcare settings offer many inherent advantages such as access to quality private services, trained manpower, efficient management of human resources, minimization of risk to both, public and private partner, better community relationships, business development or investment opportunities for private sector, sustainability for integrating private partner in public health system remain a major challenge (21).

## Conclusion

The 3Ps in India are aimed at establishing and implementing a robust PV system to monitor the safety of medicines. This approach is proven and tested and will sustain the harmonized and partnered strategy among all 3Ps stakeholders besides being cost-effective. It is expected that private sector will contribute more in near future for ensuring safe use of medicines in India.

## Data availability statement

The original contributions presented in the study are included in the article/supplementary material, further inquiries can be directed to the corresponding author.

## Author contributions

Conceptualizations, methodology, and data analysis by VK and SS. Writing—original draft preparation by VK, TS, and SS. Writing—review and editing by SS and SA. Project administration by VK and RR. All authors contributed to the article and approved the submitted version.

## Conflict of interest

The authors declare that the research was conducted in the absence of any commercial or financial relationships that could be construed as a potential conflict of interest.

## Publisher's note

All claims expressed in this article are solely those of the authors and do not necessarily represent those of their affiliated organizations, or those of the publisher, the editors and the reviewers. Any product that may be evaluated in this article, or claim that may be made by its manufacturer, is not guaranteed or endorsed by the publisher.
